# Motor-sensory biases are associated with cognitive and social abilities in humans

**DOI:** 10.1038/s41598-024-64372-2

**Published:** 2024-07-02

**Authors:** Georgina Donati, Trudi Edginton, Ameline Bardo, Tracy L. Kivell, Haiko Ballieux, Cosmin Stamate, Gillian S. Forrester

**Affiliations:** 1 Department of Psychiatry, Warneford Hospital, University of Oxford, Oxford, UK; 2 Department of Psychology, City University of London, London, UK; 3 UMR 7194-HNHP, CNRS-MNHN, Département Homme et Environnement, Musée de l’Homme, Paris, France; 4 Department of Human Origins, Max Planck Institute for Evolutionary Anthropology, Leipzig, Germany; 5 Westminster Centre for Psychological Sciences, School of Social Sciences, University of Westminster, London, UK; 6 School of Computing and Mathematical Sciences, Birkbeck, University of London, London, UK; 7 School of Psychology, University of Sussex, Brighton, UK

**Keywords:** Human behaviour, Social behaviour

## Abstract

Across vertebrates, adaptive behaviors, like feeding and avoiding predators, are linked to lateralized brain function. The presence of the behavioral manifestations of these biases are associated with increased task success. Additionally, when an individual’s direction of bias aligns with the majority of the population, it is linked to social advantages. However, it remains unclear if behavioral biases in humans correlate with the same advantages. This large-scale study (N = 313–1661, analyses dependent) examines whether the strength and alignment of behavioral biases associate with cognitive and social benefits respectively in humans. To remain aligned with the animal literature, we evaluate motor-sensory biases linked to motor-sequencing and emotion detection to assess lateralization. Results reveal that moderate hand lateralization is positively associated with task success and task success is, in turn, associated with language fluency, possibly representing a cascade effect. Additionally, like other vertebrates, the majority of our human sample possess a ‘*standard’* laterality profile (right hand bias, left visual bias). A ‘*reversed’* profile is rare by comparison, and associates higher self-reported social difficulties and increased rate of autism and/or attention deficit hyperactivity disorder. We highlight the importance of employing a comparative theoretical framing to illuminate how and why different laterization profiles associate with diverging social and cognitive phenotypes.

## Introduction

Although human cognition appears unique on the surface, it is supported by more general motor-sensory processing common across other species. While both sides of the brain are engaged across all instances of behavior, motor-sensory dominances are a fundamental principle of the two hemispheres of the vertebrate brain (see^1^). Evidence from comparative vertebrate studies demonstrates that individual-level motor-sensory biases are associated with increased cognitive capacity (e.g., measured by task performance). Furthermore, alignment of an individual’s motor-sensory bias (i.e., right vs. left) with the direction of the majority of the population confers a social benefit (e.g., group cohesion as in shoaling)^2–5^. Despite these patterns across a variety of vertebrate taxa, it is not yet clear if motor-sensory biases in the human brain influence cognitive capacity and social abilities.

Comparative research suggests that the need to carry out basic survival behaviors in parallel, like feeding while simultaneously looking out for predators, created pressure for the lateralization of adaptive brain processes in the vertebrate brain. A by-product of a divided brain is an increase in ‘*cognitive capacity*’ for the individual organism perhaps resulting from neural efficiency gained by avoiding complete duplication of functions across the left and right hemispheres^1,2,6–8^. Seminal work with domestic chicks (*Gallus gallus domesticus*) exploited the increased cognitive capacity effect via a dual task paradigm whereby chicks were engaged in a feeding task (discriminating grain from pebbles) while simultaneously monitoring for fake predators (a silhouette of a bird of prey)^9^. Compared to non-lateralized chicks, lateralized chicks (controlled by manipulating exposure to light during incubation) were faster to respond to predators when approaching from one side and were better at discriminating grain from pebbles with their right eye^10^. Task performance advantages were replicated in dual task paradigms with fishes also during feeding and predation, whereby male topminnow fish (*Girardinus falcatus)* with a turning bias were faster at catching prey and lateralized female topminnow fish were more successful at finding food, compared with their respective non-lateralized counterparts^11,12^. In marmosets (*Callithrix jacchus),* those with stronger hand preference for grasping food detected a fake predator faster than individuals with weaker hand preference^13^. In these examples, a ‘cognitive capacity’ advantage is measured by and argued to have arisen during competing tasks. However, a cognitive capacity advantage seems to be generalized for motor sequencing behavior associated with feeding in the absence of a competing task. Animals that express a limb bias, ranging from species as disparate as birds (e.g., pigeons^14^ and parrots^15^), mammals (e.g., cats^16^) and primates (e.g., chimpanzees^17^) tend to be more efficient at completing feeding tasks compared to individuals who possess comparatively weaker bias. However, the relationship between bias strength and task success may not be linear and may not extend to increased survival. For example, pheasants with stronger foot preference for feeding were more likely to die earlier than mildly lateralized individuals^18^. These studies suggest that while the presence of motor biases is beneficial for task success, there may be optimal levels of laterality that support cognitive flexibility and survival fitness.

Since animal models suggest a cognitive capacity benefit (e.g., task success) is conferred from the presence rather than the direction of motor-sensory biases, it is expected that within a population there would be equal proportions of individuals with left and right biases for the same behavior. However, motor-sensory biases frequently occur at the population level, with the majority of individuals aligning in the same direction for motor sequencing and predator detection (e.g.,^19–21^). Known as ‘population laterality’ this phenomenon is theorized to reflect an *evolutionary stable strategy* (ESS) (e.g.,^22,23^), arising from social pressures to coordinate interactions between individuals during both cooperative^2^ and competitive interactions^24^. Although some individuals may display complete laterality within a given context, notably, there is never complete laterality within a population. A minority of individuals who do not align with the majority of the population is always preserved. The reverse direction is theorized to be beneficial during competitive interactions, such as adding an element of surprise (*for an overview see*^24^) that can increase survival in the individual as well as overall survival of the population. As a result of ESS, the comparative literature reveals a prevailing ‘*standard’* pattern of motor-sensory biases across vertebrates for adaptive survival behaviors. Specifically, it is most common at the population-level to exhibit a left eye or visual field bias for monitoring threat and a right side motor bias for skilled motor action sequences (e.g., to find food) (*for a summary of vertebrates see*^22^*, also see for invertebrates*^25^). The common alignment confers many social advantages. For example, chicks with the population majority alignment were able to form stable social hierarchies gaining access to food, using a common left-eye advantage to read the hierarchical social cues of conspecifics whereas non-lateralized chicks could not^26^. In social animals, the presence of population laterality is common for maintaining group bonds for shoaling in fishes^27^, monitoring conspecifics in mammals (e.g.,^28^), navigating social spaces in primates^29^ and nurturing young in both land and sea mammals^30,31^.

Consistent with other vertebrates, tasks requiring skilled motor-action sequencing (analogous to those underpinning feeding behaviors in other vertebrates) and social stimuli processing (akin to those underpinning threat detection in other vertebrates) are two of the most robustly lateralized sensory-motor behaviors in humans (e.g.,^32^). Cross-culturally, human populations possess motor-sequencing behavior biased to the right hand (correlated with left hemisphere dominance)^33–35^, which can be demonstrated via tasks of hand skill (e.g., pegboard, *see*^36^). Additionally, selective attention, face and emotion processing (critical for threat detection) are biased to the left visual field at a population-level (associated with right hemisphere dominance)^37–40^, demonstrated via emotion detection tasks using chimeric faces (*for a meta-analysis see*^41–43^). Within the context of limb laterality, human populations exhibit population alignment even more strongly than those observed in other animal species^44^. However, it is not clear if motor-sensory biases also afford related cognitive capacity and social advantages in modern humans. Moreover, it is unclear how or if these context specific motor-sensory biases provide a foundational platform for human higher cognitive abilities, like language. Neuroscience studies demonstrate a common neural substrate underpinning structured hand action sequences and language production (e.g.,^45^), making structured manual tasks a plausible precursor and/or catalyst for the evolutionary emergence of language^46,47^. Likewise, developmental psychology argues that motor-sensory behaviors support emerging more complex, functionally-related abilities (e.g.,^48,49^) and regardless of inconsistent methods, researchers consistently report a positive association between early motor and language development (e.g.,^50^). Therefore, it is pertinent to investigate if these context-specific human motor-sensory biases confer direct or indirect cascading advantages for related higher cognitive functions.

Where bias strength is investigated, strong behavioral biases (regardless of direction) have been demonstrated in healthy child populations, consistent with the comparative literature (e.g.,^51^). However, the presence and strength of biases are lower by comparison for both motor-sequencing and emotion detection tasks in some studies of neurodiverse populations with social-communication deficits, such as autism^51–53^ and attention-deficit hyperactivity disorder (ADHD)^54^. Autism and ADHD often co-occur, commonly referred to as AuDHD, and are known to be associated with emotional processing impairments^55,56^. Populations of individuals with some neurodiverse conditions can also present differentiated patterns of motor-sensory biases compared with neurotypical counterparts (*for a meta-analysis see*^54^). Therefore, it is important to understand if and how these biases are related to cognitive and social abilities.

Studies investigating laterality and cognitive capacity in humans are scarce, inconsistent, and are not theoretically positioned within a comparative framework (*but see*^57,58^). Existing findings emanate from a mix of brain imaging, behavioral and clinical investigations and test performance on a variety of complex human cognitive functions (e.g., attention, memory, language). For example, one functional magnetic resonance imaging (fMRI) study found associations between highly lateralized language function (left or right cerebral lateralization) and higher verbal and non-verbal performance compared with non-lateralized individuals, but only weak associations between hand lateralization and language performance^59^. Some have reported no linear associations^60^, while others have found better task performance (for language and face detection tasks) in association with mild to moderate functionally-related brain lateralization, compared to absent or extreme lateralization^61,62^, suggesting over-specialisation may be detrimental to processing flexibility. Likewise, in children no consistent patterns arise from the literature associating direction of bias and cognitive ability even when neurodiverse populations are involved. A recent meta-analysis of individuals (6–19 years) revealed a significant association between non-right-handedness and reading/language impairments^63^, while a systematic review including children (ages 5.1–38.7 years) found no consistent differences in direction of handedness for autistic and non-autistic populations^64^.

There are relatively few human studies that evaluate more than one motor-sensory bias within the same individual allowing for tests of population alignment and social ability. Nevertheless, there is convincing evidence that across human populations, the majority of people exhibit motor-sequencing and emotion detection biases that are consistent with a standard profile found in other vertebrates (see for a review^40^). The ‘standard’ vertebrate brain profile plays out in humans as a left hemisphere bias for motor-sequences (akin to feeding behaviors of other vertebrates) that manifests as population-level right-handedness^65,66^, and a right hemisphere bias for detecting threat that manifests as a population-level left visual field bias for recognizing faces and emotions (*for a review see*^67^) with particularly strong results for fearful expressions^68^. Like the non-human animal literature, humans also possess a comparatively less common ‘reversed’ organisation, where functions are a mirror image of the standard profile (but motor and spatial divergence is preserved). Human literature also demonstrates that individuals can possess ‘*crowded’* profiles, where functions that would normally diverge across hemispheres are housed within a single hemisphere (left or right) (*for a review see*^40^). The high frequency of the standard profile across human populations has led to hypotheses regarding its adaptive function. However, a deeper understanding has been hampered by a lack of systematic tasks and measures used to create laterality profiles for tests for associations with cognitive and social ability (*for a systematic review see:*^69^). For example, one study reports that adults who deviate from a standard profile have poorer cognitive performance^70^, while a systematic review shows little evidence for any advantage for standard compared to non-standard lateralization profiles^71^. Children also show a population prevalence for the standard profile, but rather than providing an advantage, the reversed profile confers a social disadvantage compared with the standard and crowded profiles^72^. The reversed laterality profile occurs least frequently in neurotypical populations but increases in autistic populations by comparison for both children^72,73^ and adults^74^. To our knowledge, no human studies address if alignment of motor-sensory biases (consistent with the animal literature) with the majority of the population confers any social advantage.

Here we address unanswered questions about human motor-sensory biases and their associations with cognitive and social ability in a large, cross-sectional, heterogenous sample using both *within* and *between* participant measures. Based on the comparative literature, we hypothesize that: (1) our population will demonstrate a right hand skill bias for motor-sequencing and a left side vision bias for emotion detection; (2) the strength of hand skill bias will positively associate with both a motor-sequencing task success, and language fluency ability; (3) the majority of our sample will possess a standard motor-sensory profile that will associate with higher social skill; and (4) the reversed profile by comparison will associate with lower social skill and a higher rate of individuals with self-reported autism and/or ADHD diagnosis.

## Results

### Hypothesis 1: population biases

**Figure 1:**
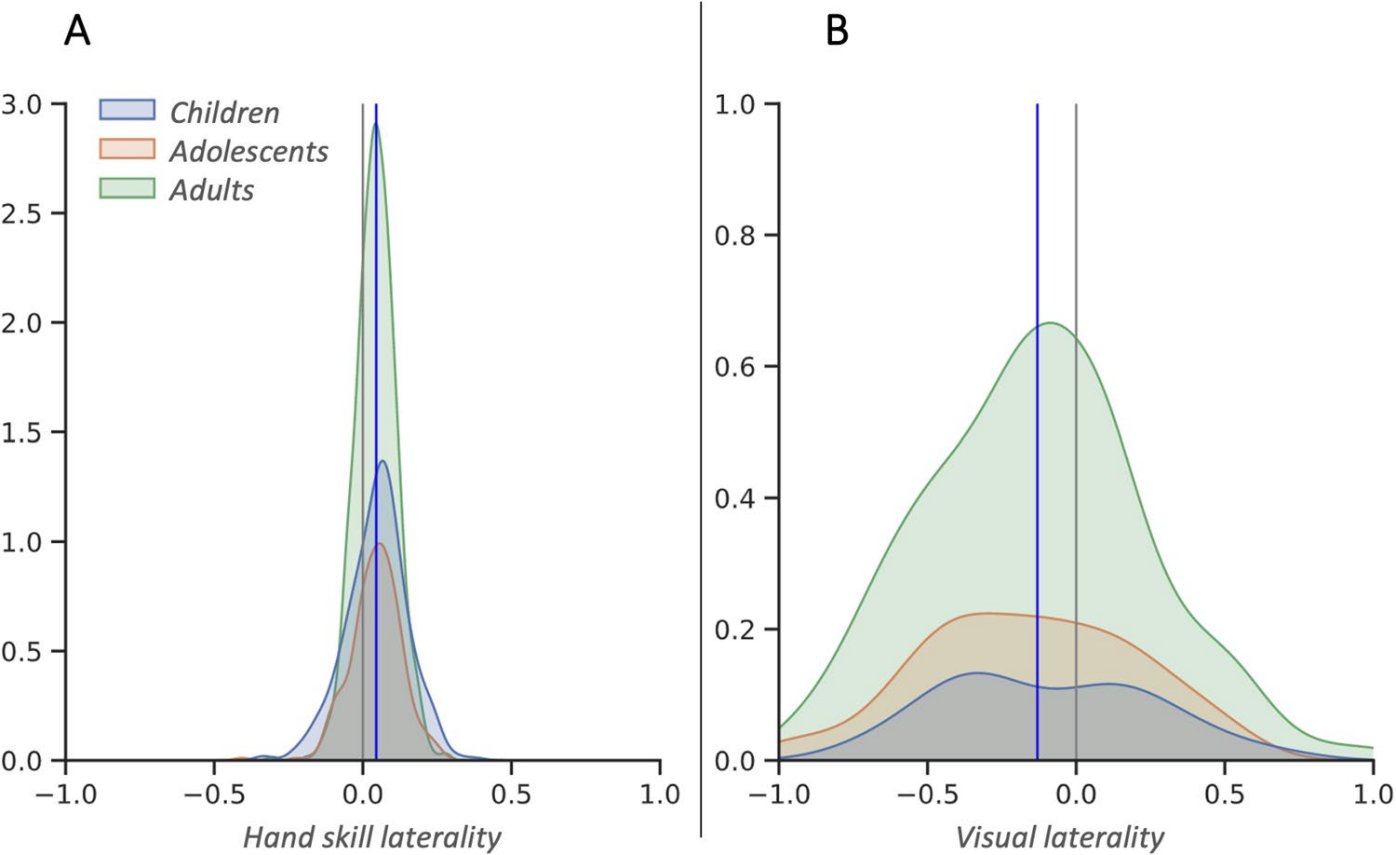
Density plots showing the distributions of (**A**) *Hand skill laterality* (measured via a motor-sequencing pegboard task) and (**B**) *Visual laterality* (measured via chimeric face emotion detection task) for children (0–10 yrs), adolescents (11–18 yrs) and adults (19 + yrs). Laterality scores range between − 1 and 1, scores below 0 indicating left dominance and scores above 0 indicating right dominance. Gray vertical line indicates 0 and blue line indicates sample mean.

We predicted that our population would demonstrate a right side hand skill bias for motor-sequencing and a left side visual bias for emotion detection. We first investigated behavioral biases in terms of the sample prevalence for sidedness, using chi-square analyses and strength of laterality using regression in *Hand skill laterality* for motor-sequencing and *Visual laterality* for emotion detection across *Age* at the population level. Overall, significantly more people were dominant with the right hand compared to the left hand for motor-sequencing (pegboard task) as would be expected (*Hand skill laterality*: *X*^2^ (1, N = 1,185 = 315.04, *p* < 0.01), but the population became less lateralized with *Age* (B = − 0.00005, *p* < 0.01)). Chimeric face stimuli, where one half of the face displays an emotion while the other side is neutral, were used to evaluate visual side bias for emotion detection. Significantly more people found faces with the expression on the left more expressive than the same expression presented on the right as would be expected (*Visual laterality*: *X*^2^ (1, N = 408) = 38.91, *p* < 0.01). This visual bias effect was stable across the lifespan (B = − 0.00003, *p* = 0.56) (Fig. 1).

### Hypothesis 2: strength of motor biases, task success and cognitive ability

We predicted that strength of *Hand skill laterality* would positively associate with hand skill ability and language fluency ability. Therefore, we tested the association of *Absolute hand skill laterality* strength (regardless of direction) with (i) motor sequencing skill (*Task success*) on a *Pegboard task* and (ii) associated score on a *Language fluency* task. Since previous literature on humans has sometimes found that a moderate laterality is the optimum for task success, we also include a quadratic term in the regression analyses. We used bootstrapping procedures to calculate robust confidence intervals to account for the non-normality of our laterality data. We found a negative quadratic association (B = − 213.22, bootstrapped CI = − 299.30 to − 134.20) between *Absolute hand skill laterality* and *Task success* (the sum of pegs placed in one minute with each hand tested separately), demonstrating moderate laterality is optimal for task success in motor sequencing actions. We found *Task success,* but not linear or quadratic *Hand skill laterality,* was associated with *Language fluency* (N = 326, (B = 0.44, bootstrapped CI = 0.33–0.56). We controlled for *Age, Sex, Maternal education and English as a first language.* Regression models explained 29% of the variance in *Task success*, 33% in *Language fluency* and 5% in *Self-reported* s*ocial difficulties* (Supplementary Table S5, *p* ≤ 0.01).

### Hypothesis 3: alignment of motor biases and social ability

**Figure 2:**
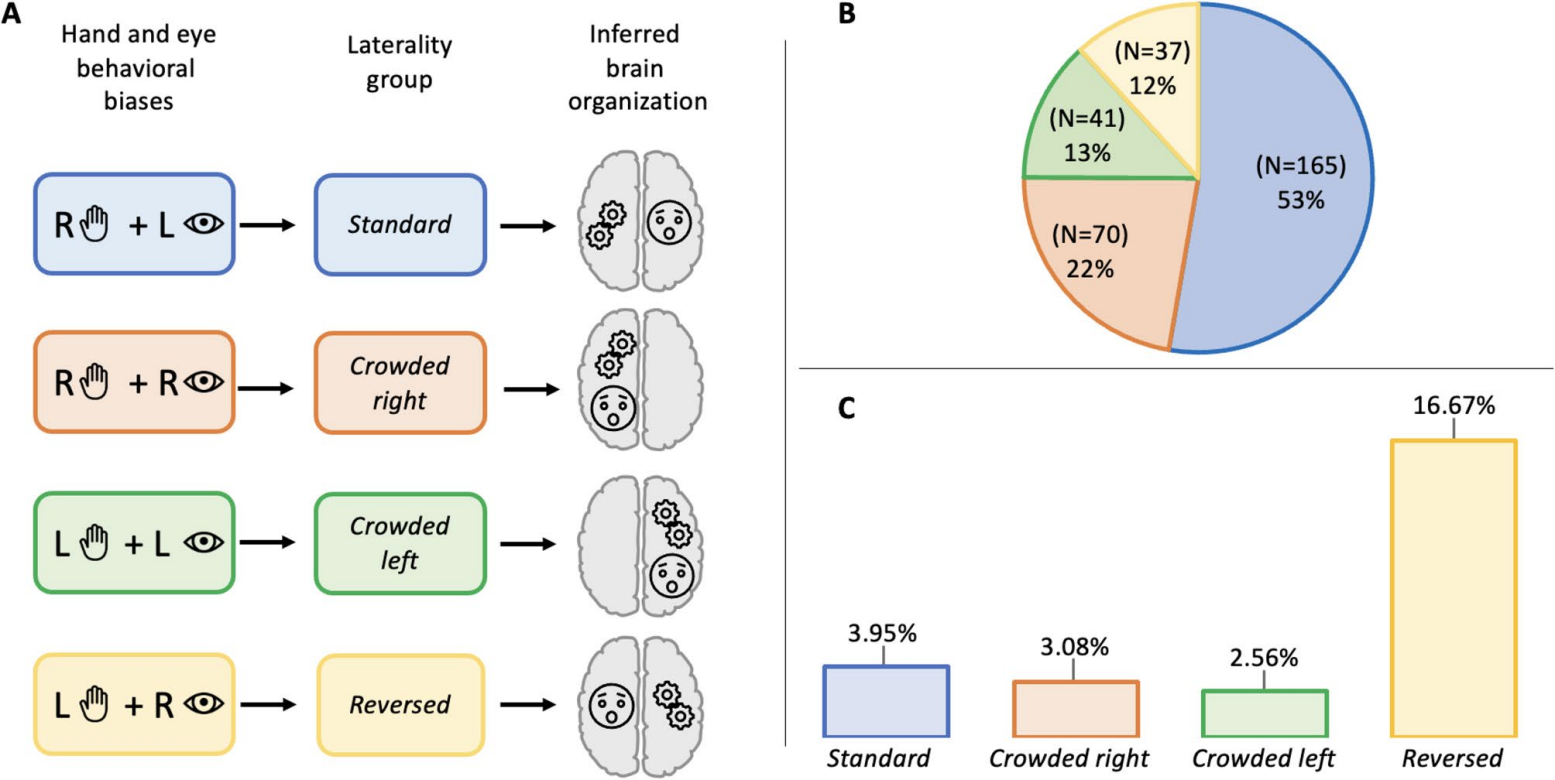
(**A**) Schema showing the relationship between behavioral biases, *Laterality group* membership and the inferred brain organisation of the group for motor-sequencing (cogs) and emotion detection (face). (**B**) The graph depicts the frequency of *Laterality group* (N = 313). Left < 0 and right > 0 however the same pattern of distribution applies when parameters are moved toward stricter thresholds e.g., left < − 0.1 and right > 0.1, N for *Crowded left* = 7, *Crowded right* = 19, *Reversed* = 7, *Standard* = 48 (*X*^2^ (3) = 55.44, *p* < 0.01) e.g., left < − 0.2 and right > 0.2, N for *Crowded left* = 0, *Crowded right* = 1, *Reversed* = 0 and *Standard* = 4 (*X*^2^ (3) = 8.60, *p* < 0.05). (**C**) The proportion of individuals *Self-reporting autism/ADHD diagnosis* in each *Laterality group.*

We predicted that the majority of our sample would possess a standard motor-sensory profile that will associate with higher social skill. To test if motor-sensory bias profiles were an existing feature of our population and if they were linked to social skill, we grouped individuals based on their dominant side for *Hand skill laterality* and *Visual laterality* scores (see Fig. 2 for thresholds) into one of four *Laterality groups*: *Standard* (right hand skill bias—left vision bias), *Reversed* (left hand skill bias—right vision bias), *Crowded right* (right hand skill bias—right vision bias), *Crowded left* (left hand skill bias—left vision bias). The most common profile was the *Standard* motor-sensory bias profile, which described 53% of the population. *Reversed* profile occurred least frequently at 12%. We also found that *Crowded Right* is more common that *Crowded Left*. A chi-square test of good fit found this distribution deviated significantly from the null hypothesis (*X*^2^ (3) = 136.52, *p* < 0.01) (N = 313) (Fig. 2).

We performed an ANCOVA to assess the differences between *Laterality groups* and *Self-reported social difficulties*. We covaried for *Age*, *Sex*, and *Maternal education* and allowed for interactions where results violated the homogeneity of regression slopes. *Self-reported social difficulties* (N = 269) revealed a significant main effect of *Laterality group* (F(3, 251) = 3.87, *p* = 0.01, η2 = 0.04) driven by a difference between the *Standard* and *Reversed* profiles (*p* < 0.05) and the *Crowded right* and *Reversed* profiles (*p* < 0.01). A significant main effect of *Age* (F(1, 251) = 8.13, *p* < 0.01, η2 = 0.03) and a significant interaction between *Laterality group* and *Sex* (F(3, 251) = 2.87, *p* < 0.05, η2 = 0.03) and *Laterality* Group and *English as a first language* (F(3, 251) = 2.72, *p* < 0.05, η2 = 0.03) were also detected. Neither *Sex* (F(1, 251) = 2.21 , *p* = 0.14) nor *Maternal education* (F(5, 251) = 1.19, *p* = 0.32) were significant. Because the main effect of *Laterality group* was driven by the difference between *Reversed* and two right-handed groups (*Standard* and *Crowded right*), we tested to ensure this was not just a right-handed effect. Results demonstrated that *Hand laterality* group was not a significant predictor of *Self-reported social difficulties* (N = 889, F(1, 878) = 2.14, *p* = 0.15).

### Hypothesis 4: alignment of motor biases and autism/ADHD

**Figure 3:**
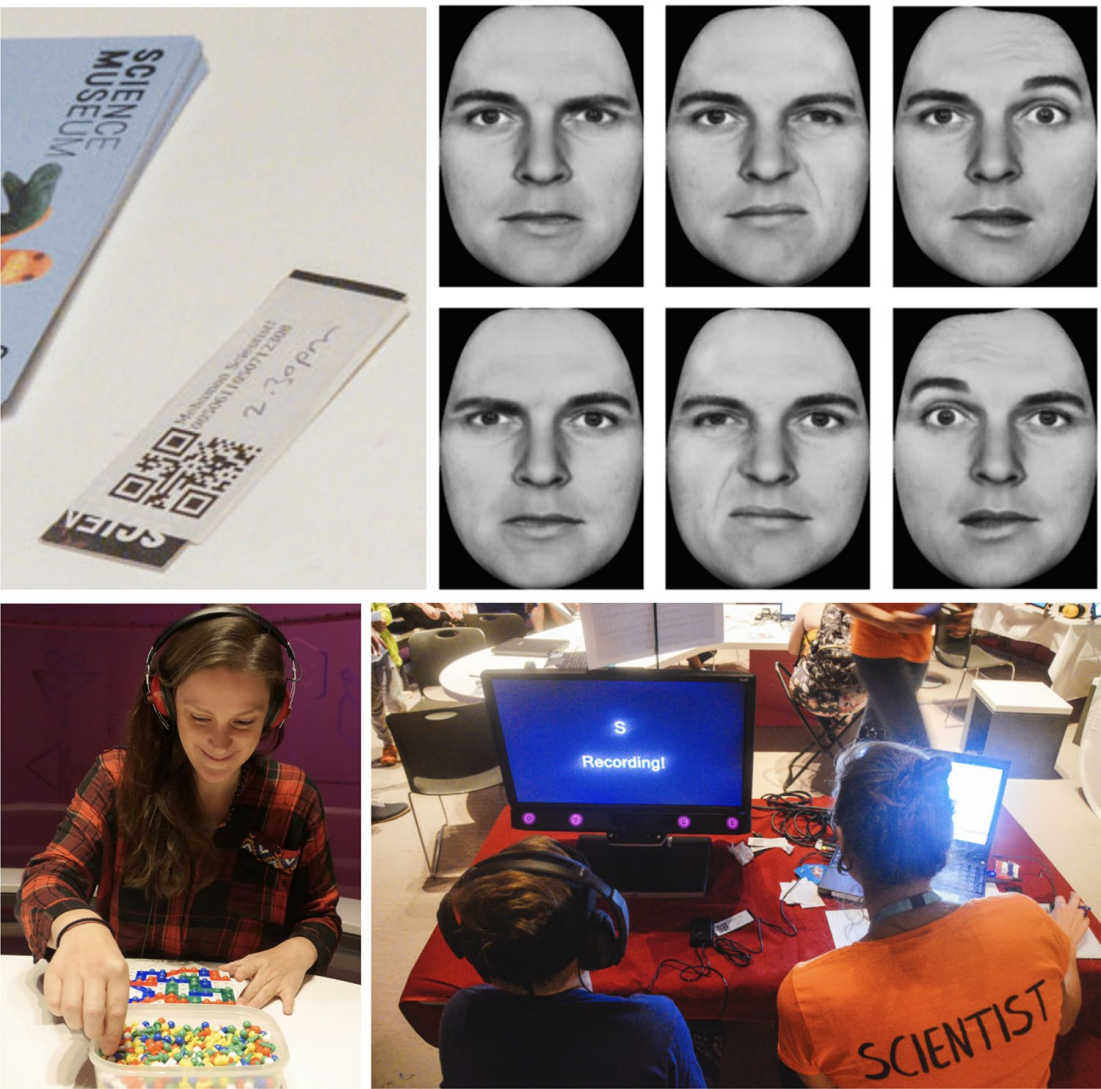
After providing informed consent and demographic information, all participants received a unique QR code (top left), which allowed them to participate in tasks guided by an experimenter. The strength and direction measures of *Hand skill laterality* were derived from a pegboard task secured to the table (bottom left), while *Task success* was evaluated by summing the number of pegs placed by each hand separately under a time constraint. *Self-reported social difficulties* scores were derived from the Autism Quotient survey^90^ which were administered using electronic tablets. *Visual laterality* scores for emotion detection bias were calculated using chimeric face stimuli^91^. Examples of fear, disgust and surprise are provided (top right). Participants engaged in the chimeric face task and language fluency tasks in front of a monitor and audio responses for the language fluency task were recorded electronically for transcription (bottom right). Photo credits (bottom left and right panels): J. Beijinho.

Finally, we predicted that the *Reversed* profile, by comparison to the other profiles, would have lower social skill and a higher rate of individuals with self-reported autism and/or ADHD diagnosis. To test this hypothesis, we performed a chi-square test to assess the representation of *Self-reported autism/ADHD diagnosis* in our *Laterality group* and found there to be a significant difference between groups (N = 292, *X*^2^ (3) = 11.358, *p* = 0.01 with *Crowded left* = 1/39, *Crowded right* = 2/65, *Reversed* = 6/36 and *Standard* = 6/152) (See Fig. 3A below). To establish whether this was specific to autism/ADHD rather than a feature of neurodiversity in general, we re-ran the analysis including all self-report neurodiversity including: autism, ADHD, developmental coordination disorder, dyspraxia, dyslexia and obsessive–compulsive disorder. We found no significant difference in group numbers; (N = 292, *X*^2^ (3) = 5.745, *p* = 0.125 with *Crowded left* = 2/39, *Crowded right* = 4/65, *Reversed* = 7/36, and *Standard* = 16/152). Although sample sizes were very small, we performed a chi-square test to assess the representation of only self-reported autism diagnosis across *Laterality group* and found there was a significant difference between groups (N = 292, *X*^2^ (3) = 9.2381, *p* = 0.026 with *Crowded left* = 0/39, *Crowded right* = 0/65, *Reversed* = 3/36 and *Standard* = 3/152).

## Discussion

Like the comparative literature, our findings show a robust right side hand skill bias for a motor sequencing task and a left side visual bias for an emotion detection task. We also found that the strength of *Absolute hand skill laterality* for motor-sequencing is associated with increased *Task success*, regardless of direction. However, we find a negative quadratic relationship whereby both weaker and stronger bias is associated with poorer cognitive performance compared with moderate laterality. This finding is consistent with previous studies in humans (e.g.,^61,62^) (and contrasts the animal literature (e.g.,^17^), demonstrating moderate laterality is optimal for task success in motor sequencing actions. It is possible that extreme bias inhibits necessary levels of interhemispheric communication for healthy cognition and decreases cognitive flexibility. Contrary to our prediction, the strength of *Absolute hand skill laterality* is not associated with *Language fluency*. Instead, *Hand skill laterality* is only associated with *Task success* and *Task success* was only associated with *Language fluency*. These relationships present the possibility of a ‘cascade’ effect akin to that proposed in developmental psychology whereby basic motor-sensory behaviors support more complex, functionally-related abilities, but can also highlight significant and valuable associations about the integrity of the cognitive developmental trajectory^71,75^. The cascade effect may also be why some previous studies find that handedness is only weakly related to language performance (e.g.,^59^), although this explanation requires further testing within a developmental context. These results suggest that human motor biases (when consistent with motor sequencing biases for adaptive survival behaviors in other animals), may confer task performance advantages that have cascading advantages for related higher cognitive functions, perhaps the result of efficient brain organization (e.g.,^3^). However, humans demonstrate a ‘goldilocks effect’ where moderate lateralization is beneficial (i.e., not too little and not too much). This could explain the mixed results in previous human studies and could differ from other species as a result of increased lateralization in humans. We could not test strength of *Visual laterality* with a visual test of success because there was no ‘right’ or ‘wrong’ answers for the chimeric face task. In future studies, an emotion discrimination task may allow testing for a cascade effect. However, since all animal literature tests ‘cognitive advantage’ only in relation to motor sequencing tasks, it is unknown if emotion detection bias relates to social-cognitive ability from a comparative perspective.

The ‘walk-in’ nature of our participant sample resulted in a decreased population for within-participant analyses. Nevertheless, there was a clear and robust population majority for a *Standard* bias profile consistent with the comparative literature^7^ and recent reports in children^72^ and adults^40^. The prevalence for a *Standard* profile emerges despite research that suggests that biases may develop independent of each other with regard to both strength and direction^4^ and supports the presence of an ESS^23^. We also find, as in previous human research, that Crowded Right is more common that Crowded Left and Reversed is least common (e.g.,^72^). We find some evidence that *Laterality group* is associated with social ability, driven by higher *Self-reported social difficulties* in the *Reversed* bias profile compared with the *Standard* and *Crowded right* bias profiles. In our human sample we have more complex laterality profiles, that include crowded organizations, compared with the comparative animal literature. As such we are able to establish that in humans, the *Standard* profile alone is not associated with better social ability. In fact, there are comparable social scores across the *Standard* and *Crowded* profiles. Rather it is the *Reversed* profile that is associated with greater social difficulties. This finding is further supported by the significantly higher rate of *Self-reported autism/ADHD diagnosis* in the *Reversed* group, conditions known to be characterized by differences in social processing^76^. An additional analysis revealed that, consistent with recent reports (e.g.,^77,78^), there was no association between *Laterality group* and diagnosis for other self-reported neurodiverse conditions, further supporting the hypothesis that it is the social symptomatology of autism and ADHD that drives the effect. Members of the *Reversed* profile group are not aligned with the population majority for both motor-sequencing and emotion detection tasks. It is possible that this double misalignment impacts the timing of the comprehension and production of social cues that in turn may disrupt the temporal synchrony required for fluid social engagement (*see for a review*^79^).

These results regarding bias direction are important because evidence from studies involving children suggests that biases for motor-sequencing and emotion detection can be visible from a young age^72,80–83^ and are unlikely to change in direction during development^73^, suggesting these biases may develop early in ontogeny. Our findings, therefore, have implications for autism research where motor-sensory differences are often cited comorbidly with social and communication differences during development, compared with neurotypical counterparts^49,84–86^. Laterality bias profiles may act as an early marker for individuals with risk for these conditions, providing a new window on development and the potential for innovations in early interventions to improve cognitive outcomes for at risk infants. They may also help us make sense of windows where early bias patterns are already visible within the literature. For example, results from recent infant eye-tracking studies show retrospectively that 6-months-old neurotypical infants looked at face stimuli equally on the left and right side of space whereas infants who subsequently received an autism diagnosis showed a lateralized preference for looking at faces on the right and were slower to look at faces on the left. At 14 months of age, this difference was no longer visible despite these infants going on to receive an autism diagnosis^87^. A similar pattern is visible in 8–10-month-old preterm infants who showed reduced interest to social stimuli on the left side of space compared with their full-term counterparts^88^. These studies suggest that visual biases for social stimuli may be shaped during development resulting from both biology and environment. Moreover, studies involving conditions that are characterized by differences in social processing, like autism, may be better suited to testing bias alignment, over bias strength, as the condition does not necessarily impair other cognitive functions and may in some cases create advantages.

It is important to note that this study provides very restricted measures to test hypotheses specifically aligned with the comparative literature (motor action and emotion processing). As such, we can only interpret our findings within this narrow, but theoretically grounded context. We understand that there are many and more complicated ways to categorize laterality profiles, measure task success and evaluate cognitive ability. We also acknowledge that individual biases are not necessarily correlated, such that an individual may have one laterality profile under one set of measures and a different profile under another. It is possible that a lack of robust patterns emerging from the human literature is due to a reliance on complex, high-level human-orientated abilities without the integration of grounded theoretical comparative models to help us understand the relationships between more basic motor-sensory biases and higher-level cognition.

## Conclusion

Systematic and replicable approaches are required to build new hypotheses that can reveal the key factors shaping human cognitive abilities. Employing a comparative theoretical framing to investigate the strength and directional alignment of human motor-sensory biases may illuminate how and why early laterization may lead to diverging social and cognitive phenotypes and what the advantages of this may be for both the population and the individual. We do not yet know if human behavioral biases evolved from basic patterns of lateralization common to all vertebrates^7^ or emerged from processes related to coevolution^89^, at the population level, these biases are present and robust in humans. However, it is important to acknowledge that in the same way that our psychological explanations must be consistent with biology, they must also align with evolution. This challenge can be most effectively tackled by looking comparatively across species. A comparative lens affords a broader understanding of *how* and *why* some human motor-sensory and more complex skills are supported in an organisationally biased fashion. Comparative literature distinguishes between the role of lateralization in an individual and in a population. Both have their advantages but for different adaptive reasons. Our results demonstrate some continuity with the animal literature as well as some new findings that together suggest that for humans (1) moderate individual lateralization is associated with better task performance with a potential cascade to higher cognitive functions; and (2) reversed alignment compared with the population majority is associated with higher self-reported social difficulties and self-reported diagnosis of autism and/or ADHD. Understanding why these bias patters occur and their influence on cognitive and social abilities is important for future research and may provide new approaches to investigate risk in developing cognition.

## Methods

All methods were performed in accordance with the relevant guidelines and regulations of the 1964 Declaration of Helsinki. Ethical approval for the current study was authorized by the Department of Psychological Sciences Ethics Committee at Birkbeck (ref: 181,996), University of London. Participants (n = 313–1661 depending on analysis, see Supplementary Table S1) were opportunity sampling visitors to The Science Museum, London, during a 3-months Live Science summer residency 2019. Participants completed a demographic questionnaire from which we created the variables *Age*, *Sex, Maternal Education, English as a first Language* and *Self-reported autism/ADHD diagnosis* (see Supplementary Table S2). Lateral biases were measured using a pegboard task and chimeric face task (see Supplementary Table S3) to determine individual bias strength and population alignment. These tasks were relevant because not only do they align with the context of adaptive survival behavior from the comparative literature, they are robustly measured via motor-sensory behaviors (*for an overview see*^1^), which are arguably more powerful than neural imaging techniques because fundamentally it is behavior that results in survival. *Task success* was measured via success on the pegboard task (total number of correct pegs placed with both hands) and cognitive ability was measured via phonemic *Language fluency* performance, while *Self-reported social difficulties* were measured via the autism quotient questionnaire (see Supplementary Tables S3 and S4). See Fig. 3 for an example of the tasks undertaken by participants. Informed consent was obtained for publishing identifying images in an online open-access publication. Absolute laterality scores were used in three regression models using bootstrapping (2000 iterations and the R *boot* package) and included *Age*, *Sex*, *Maternal education and English and a first Language* as covariates to evaluate bias strength and cognitive performance (see Supplementary Table S5). Individuals were grouped, based on their dominant side for *Hand* and *Eye*, into one of four *Laterality Groups*: ‘*Standard’* (right hand skill bias—left visual side bias), ‘*Reversed*’ (left hand skill bias—right visual side bias), ‘*Crowded right*’ (right hand skill bias—left visual side bias), ‘*Crowded left*’ (left hand skill bias—left visual side bias) and two ANCOVAs were used test our hypotheses that a standard profile would be advantageous for social abilities.

### Supplementary Information

Supplementary Information.

## Data Availability

All data required to evaluate the results in the paper are present in the paper and/or in the Supplementary Materials and are also available via the Open Science Framework: https://osf.io/b285y/.
