# Supplementary material for: Motor-sensory biases are associated with cognitive and social abilities in humans
Source: Sci Rep. 2024 Jul 2;14:14724. doi: 10.1038/s41598-024-64372-2 (PMC11219847; doi:10.1038/s41598-024-64372-2)
Supplement: Supplementary file 1 — Supplementary Information. [file 41598_2024_64372_MOESM1_ESM.pdf]

Supplemental Materials:

Motor-sensory biases are associated with cognitive and social abilities in humans

Georgina Donati[1,6†], Trudi Edginton[2], Ameline Bardo[3,4], Tracy L. Kivell[4], Haiko Ballieux[5], Cosmin Stamate[6], Gillian S Forrester[7†]*

Affiliations:

[1]Department of Psychiatry, University of Oxford; Warneford Hospital, Oxford, UK

[2]Department of Psychology, City University of London, London, UK

[3]UMR 7194-HNHP, CNRS-MNHN, Département Homme et Environnement, Musée de l'Homme, Paris, France

[4]Department of Human Origins, Max Planck Institute for Evolutionary Anthropology, Leipzig, Germany

[5]Westminster Centre for Psychological Sciences, School of Social Sciences, University of Westminster; London, UK

[6]School of Computing and Mathematical Sciences, Birkbeck, University of London; London, UK

[7]School of Psychology, University of Sussex, Brighton, UK

([†]equal contribution by first and last authors)

*Corresponding author Email: g.forrester@sussex.ac.uk

Supplemental Materials:
Materials and Methods
Table S1 – S5
References [82, 90-94]

Materials and Methods

Participants

Participants were opportunity sampling visitors to The Science Museum, London, during a 3-months Live Science summer residency 2019. Experiments were performed in a closed off section of the Wellcome Trust's 'Who Am I?' gallery and members of the public were invited in to participate. Participants came from all around the world and experiments were devised so that the ability to speak English was not required to participate. An international group of 24 researchers were able to explain basic rules to any individuals who did not speak English. All participants gave informed consent or legal guardians gave consent for those under 18 years of age via a digital consent form presented on a tablet with tick box. On arrival participants were given a code allowing them to participate anonymously in as many of the experiments as they wanted. The original sample consisted of 1708 participants. Participants were excluded from the following sample if they had any physical diagnoses that would impede participation in the tasks, including visual and auditory impairment or self-report brain damage impairing cognitive ability (**Supplementary Table S1**). Based on the above exclusion criteria, three individuals were removed due to brain damage impairing task performance (e.g. stroke) and 42 individuals were excluded due to physical diagnosis impairing task performance (e.g. visual impairment). Two additional individuals were excluded for not placing any pegs with the second hand. The remaining dataset included 1,661 participants.

Supplementary Table S1: Descriptive statistics of the sample

Sample	N	% of group	Female N	Age range in years(yrs)/months(mos)	Mean age in months
0 – 10	500	30.1	267	9 mos – 10yrs,11mos	7yrs, 6mos
11 – 18	330	19.9	186	11 yrs – 18 yrs,11mos	14yrs, 6mos
19 +	831	50.0	504	19 yrs – 82 yrs,6 mos	36yrs, 7mos
Full sample	1661	100	958	9 mos – 82 yrs,6 mos	24yrs, 2mos
Laterality group	313	18.8	179	5 yrs,7 mos – 81 yrs,8 mos	26yrs, 9mos

Supplementary Table S2: Sample characteristics

Maternal Education (highest completed)		Sample N	%
	Primary School	75	5%
	Secondary School	415	26%
	Technical qualification	238	15%
	Bachelor degree	502	32%
	Masters degree	280	18%
	Doctorate	76	5%
English as a first Language			
	Yes	1057	67%
	No	604	33%
Autism/ADHD			
	No	1471	96%
	Yes	62	4%
Neurodiverse			
	No	1399	91%
	Yes	135	9%

Questionnaire:

Participants completed a demographic questionnaire from which we created the variables *Age*, *Sex, Maternal Education, English as a first Language, Self-reported Autism/ADHD* diagnosis and *Neurodiversity.*

Hand skill laterality: Pegboard task

Hand skill laterality was measured using the pegboard task [modified from 1]. The participant was positioned in front of a peg board (10 x 10 holes) with a bowl of multi-colored pegs centrally behind the pegboard. The pegboard was colored with red, green and blue lines and the participant was required to match the peg to the color on the pegboard. The bowl with the pegs included white and yellow distracter pegs. Participants were challenged to match as many pegs as possible to the corresponding color in the board in one minute using only one hand and picking out only one peg at a time from the bowl with that one hand. They then performed the same task with the other hand. First-hand use was counterbalanced over participants to account for any training effects. Scoring each hand separately was chosen over allowing participants to use both hands to facilitate coding and prevent 'cheating' (picking up more than one peg at a time). A classic laterality score was calculated (right - left / right + left) using the number of pegs correctly placed by each hand to evaluate population-level bias and categorize individuals into *Laterality group*. *Absolute hand skill laterality* scores were calculated in the same fashion disregarding direction to test associations with *Task* success. A *Task success* score was created by adding together total number of pegs successfully placed by both hands (one minute per hand) **Supplementary Table S3**).

Supplementary Table S3: Descriptive statistics for each variable used in the analysis

Variable	Age range (months)	N	M	Range	SD
Hand skill laterality	35-990	1321	0.045	-0.41 - 0.39	0.08
Visual laterality	63-980	458	-0.14	-1 - 1	0.37
Absolute Hand skill laterality	35-990	1321	0.08	0 - 1	0.06
Absolute visual laterality	63-980	458	0.31	0 - 0.14	0.23
Task success	35-990	1321	45.5	10 - 72	10.4
Language fluency	63-980	390	25.47	3 -54	9.39
Self-reported social difficulties	40-990	1290	3.18	0 - 11	2.34
Laterality Group	67 - 980	313	-	-	-

N = number of participants in sample, M = mean, SD = standard deviation

Visual laterality: Chimeric face task

This task was developed in line with Innes and colleagues [2], to assess visual field biases for processing emotional information. The paradigm utilizes chimeric faces split vertically down the middle with half of the face presenting an emotion and the other half a neutral expression. The faces with the same emotional expression, on opposites sides are then presented on top of each other briefly to the participant who is required to report which face they find more expressive. This study used three different negative emotional expressions to elicit threat response: angry, disgust and surprise, along-side a neutral expression. Participants were seated approximately 60cm from the screen and stimuli were presented in pairs, one above the other (horizontal visual angle 25° 5' 0.28"; vertical visual angle per face 35° 13' 0.76"), followed by a fixation cross. Each expression was presented six times, three where the expression was to the left on the top and three where it was to the left on the bottom. Every order combination of faces was separated by a neutral trial i.e., Angry-top-left; Disgust-bottom-left; Surprise-top-left; Neutral; Angry-bottom-left; Disgust-top-left; Surprise-bottom-left; Neutral; Surprise-top-left; Angry-top-left; Disgust-bottom-left; Neutral; Surprise-bottom-left; Angry-bottom-left; Disgust-top-left; Neutral; Disgust-bottom-left; Surprise-top-left; Angry-top-left; Neutral; Disgust-top-left; Surprise-bottom-left; Angry-bottom-left; Neutral. In each trial the fixation cross was presented for 600ms. followed by the presentation of a face pair for 4000ms. The participant was asked to state which face was more expressive, top or bottom. If no answer was given it was counted as a missed trial. A classic laterality score was calculated (right - left / right + left) using the number of reported trials in which the participant reported the face with the expression on the left or right was more expressive used to evaluate population-level bias and categorize individuals into *Laterality* group. *Absolute visual laterality* scores were calculated in the same fashion disregarding direction to test associations with *Self-reported social difficulties*, **Supplementary Table S3**).

Language fluency: Phonemic verbal fluency task

To assess language fluency, we adapted the F-A-S Test, a subtest of the Neurosensory Center Comprehensive Examination for Aphasia [NCCEA, 3]. In this study participants were given one minute to verbally express, in English or their native language, as many words as they could think of starting with the letter that appeared on the screen in front of them. They were instructed that they could say any words but that proper nouns would not be counted. This was then repeated with a second letter. The two letters used were ‘S’ and ‘L’ with order counterbalanced across collection days. Previous studies [e.g., 4] have shown ‘L’ to be a difficult letter and ‘S’ an easier letter based on the frequency of words in the English language beginning with these letters. The session was recorded and audio-transcribed for scoring. Participants were given a point for each word not including repetitions, errors (e.g., the inclusion of words that did not begin with that letter or proper nouns) or variations on the same word-base, i.e., ‘sag’ and ‘saggy’. Scores for L and S were added together to produce an overall Language fluency score (N = 390, M = 25.47, Range = 3 – 54).

Self-reported social difficulties: Autism Quotient

A social-communication combined score was created from the social and communication scales of the Autism Quotient (AQ) [5] (**Supplementary Table S4**). Six questions from each of the social, communication and attention scales were used. Scale scores were added together to create a score ranging from 0 – 12 where higher scores represented greater social-communication difficulties. The communication scale of the AQ measures social aspects of communication relating to understanding social cues from communication partners (e.g., ‘People often tell me that I keep going on and on about the same thing’; ‘I enjoy social chit-chat’; I know how to tell if someone listening to me is getting bored’). We considered these examples of ‘communication’ to represent the ability to read social

cues via responding to the direction and level of attention as well as the emotive states of social partners, rather than expressive or receptive language ability. As such, we deemed these abilities, like the social questions, to be right-hemisphere dominant traits for the majority of the population and different from the language fluency scores which we expected to represent a left hemisphere language function (N = 1,290, M = 3.18, Range = 0 – 11).

Analysis

Data cleaning and analyses were all performed in R version 4.0.2 [6]. Individual with self-report hearing or visual impairment were removed from analysis as well as those who had a stroke or other cognitive injury resulting from brain damage and/or memory impairment. First, at a population level, dominances in terms of *Hand skill laterality* and *Visual laterality* were looked at across domains, *Age* and *Sex*. Next absolute laterality scores were used in three regression models using bootstrapping (2000 iterations and the R *boot* package) to estimate robust confidence intervals to test hypotheses with regard to individual level laterality. Robust regression was used to deal with the non-normal distribution of residuals. Our hypotheses were that strength of *Hand skill laterality* would predict *Task success*, *Hand skill laterality* and *Task success* would predict *Language fluency*, an associated cognitive task, and that *Visual laterality* would predict *Self-reported social difficulties*. Covariates included *Age*, *Sex*, *Maternal education and English and a first Language* as we expected these to predict out outcome measure in a way that might mask any effect of laterality. Finally, individuals were grouped, based on their dominant side for *Hand skill laterality* and *Visual laterality*, into one of four *Laterality Groups*: '*Standard'* (right hand skill bias - left visual bias), '*Reversed*' (left hand skill bias- right visual bias), '*Crowded right*' (right hand skill bias - left visual bias), '*Crowded left*' (left hand skill bias – left visual bias). We performed two ANCOVAs to test our hypotheses that a standard profile will be advantageous for social abilities and the reversed profile disadvantageous. We did not expect *Laterality Group* to be associated with *Language Fluency* and include it as a control. We covaried for *Age*, *Sex*, *Maternal education* and *English as a first Language* analysis and allowed for interactions where results violated the homogeneity of regression slopes. To deal with the non-normal distribution of residuals in the *Self-reported social difficulties* ANCOVA due to a positively skewed *Self-reported social difficulties* measure, this measure was rank based normalized using the *RankNorm* function in the *RNOmni* R package.

Supplementary Table S4: Autism Quotient survey for social (SOC) and communication (COMM) skills.

Question	Scale
People often tell me that I keep going on and on about the same thing.	COMM
Other people frequently tell me that what I've said is impolite, even though I think it is polite.	COMM
I enjoy social chit-chat.	COMM
I frequently find that I don't know how to keep a conversation going.	COMM
I know how to tell if someone listening to me is getting bored.	COMM
I am often the last to understand the point of a joke.	COMM
I prefer to do things with others rather than on my own.	SOC
I find social situations easy.	SOC
I find myself drawn more strongly to people than to things.	SOC
I find it easy to work out what someone is thinking or feeling just by looking at their face.	SOC
I enjoy social occasions.	SOC
I enjoy meeting new people.	SOC

Supplementary Table S5: Full multiple regression results with standard and robust confidence intervals.

		b	95%CI	bootstrapped 95%CI
Task success				
	Hand skill laterality Quadratic	-213.22*	-302.93, -123.50	-299.30, -134.20
	Hand skill laterality Linear	10.60	-10.65, 31.85	-8.90, 32.25
	Age in months	0.02*	0.02, 0.02	0.02, 0.03
	Sex	3.39*	2.40, 4.38	2.43, 4.44
	Maternal Education[1]			
	Linear	2.96*	0.86, 5.06	0.98, 4.92
	Quadratic	-1.44	-3.38, 0.50	-3.22, 0.28
	Cubic	0.80	-0.73, 2.33	-0.67, 2.17
	English as a first lang	-2.42*	-3.47, -1.36	-3.45, -1.37
Language fluency				
	Task success	0.44*	0.32, 0.56	0.33, 0.56
	Hand skill laterality Quadratic	128.94	-84.27, 342.16	-63.90, 308.20
	Hand skill laterality Linear	-13.90	-58.17, 30.36	-58.48, 28.07
	Age in months	0.02*	0.02, 0.03	0.02, 0.03
	Sex	-0.47	-2.30, 1.35	-2.5, 1.36
	Maternal Education[1]			
	Linear	5.97*	1.89, 10.05	1.96, 10.20
	Quadratic	-2.03	-5.72, 1.67	-5.78, 1.75
	Cubic	-1.17	-4.11, 1.78	-4.28, 1.89
	English as a first lang	3.98*	2.08, 5.87	2.08, 5.81
Self-reported social difficulties				
	Visual laterality Quadratic	-0.9	-4.52, 2.59	-4.12, 2.46
	Visual laterality Linear	0.58	-2.47, 3.63	-2.52, 3.40
	Age in months	-0.002*	-0.004, -0.001	-0.004, -0.001
	Sex	-0.65*	-1.13, -0.17	-1.1508, -0.16
	Maternal Education[1]			
	Linear	-0.56	-1.66, 0.54	-1.65, 0.56
	Quadratic	1.06	0.08, 2.05	0.05, 1.99
	Cubic	0.36	-0.42, 1.14	-0.49, 1.16
	English as a first lang	-0.52	-1.01, -0.03	-1.04, -0.04

* <- 0.01, [1]Maternal education is entered as an ordered factor and so linear, quadratic and cubic effects are calculated.

References

1. Forrester, G. S., Davis, R., Mareschal, D., Malatesta, G. & Todd, B. K. The left cradling bias: An evolutionary facilitator of social cognition? *Cortex* **118**, 116–131 (2019).
2. Innes, B., Burt, D. M., Birch, Y. & Hausmann, M. A leftward bias however you look at it: Revisiting the emotional chimeric face task as a tool for measuring emotion lateralization. *Laterality* **21**, 643–661 (2016).
3. Spreen, O. & Benton, A. L. *Neurosensory Center Comprehensive Examination for Aphasia: Manual of instructions (NCCEA)* (rev. ed.). (Victoria, BC: University of Victoria, 1977).
4. Barry, D., Bates, M. E. & Labouvie, E. FAS and CFL forms of verbal fluency differ in difficulty: a meta-analytic study. *Appl. neuropsychol.* **15**(2), 97–106 (2008).
5. Baron-Cohen, S., Wheelwright, S., Skinner, R., Martin, J. & Clubley, E. The Autism-Spectrum Quotient (AQ): Evidence from Asperger syndrome/high-functioning autism, males and females, scientists and mathematicians. *J. Autism Dev. Disord.* **31**(1), 5–17 (2001).
6. R Core Team. R: A Language and Environment for Statistical Computing. R Foundation for Statistical Computing, Vienna, Austria. URL http://www.R-project.org/ (2013).
